# Effects of Ghrelin on germ cell apoptosis and proinflammatory cytokines production in Ischemia-reperfusion of the rat testis

**Published:** 2015-02

**Authors:** Majid Taati, Mehrnoush Moghadasi, Omid Dezfoulian, Payman Asadian, Morteza Zendehdel

**Affiliations:** 1 *Department of Pathobiology, School of Veterinary Medicine, Lorestan University, Khorramabad, Iran.*; 2 *Department of Physiology, Faculty of Medicine, Lorestan University of Medical Sciences, Khoramabad, Iran.*; 3 *Department of Physiology, Faculty of Veterinary Medicine, University of Tehran, Tehran, Iran.*

**Keywords:** *Ghrelin*, *Ischemia*, *Reperfusion*, *Testes*, *Apoptosis*

## Abstract

**Background:**

Testicular torsion is a medical emergency that requires surgical intervention to reperfuse the affected testis. Ischemia reperfusion injury is usually associated with proinflammatory cytokine generation and apoptosis of germ cells in the testes.

**Objective:**

In this study we investigate the effect of ghrelin on the proinflammatory cytokines levels and germ cell apoptosis in testicular ischemia reperfusion.

**Materials and Methods:**

45 male rats were selected for the study and randomly divided into 3 groups, each containing 15 rats. Animals in the testicular torsion and ghrelin treated groups were subjected to unilateral 720 counterclockwise testicular torsion for 1 hr and then reperfusion was allowed after detorsion for 4 hr, 1 and 7 days. The ghrelin-treated group received intraperitoneal injection of ghrelin 15min before detorsion. The expression levels of bcl-2-associated X protein and proliferating cell nuclear antigen in testicular tissue in the different groups were detected by immunohistochemical assay and tissue cytokines interleukin-1β, tumor necroses factor-α and interleukin-6 were measured using enzyme-linked immunosorbent assay

**Results:**

After being treated by ghrelin, the population of immunoreactive cells against BAX in the spermatocytes on day 7 after reperfusion significantly decreased when compared to tortion/ detortion-saline animals (p=0.024). In contrast, PCNA expression in the spermatocytes and spermatogonia were not significantly different between tortion/ detortion-ghrelin and tortion/ detortion-saline groups on both experimental days. Administration of ghrelin significantly attenuated the testicular tumor necroses factor-α and interleukin-6 levels compared with the untreated animals, but had no significant effect on the level of interleukin-1β.

**Conclusion:**

Ghrelin offers remarkable anti-inflammatory and anti-apoptotic effects in testicular ischemia reperfusion injury.

## Introduction

Testicular torsion is a medical emergency that results in an obstruction of blood flow to the testis and usually requires surgical intervention to reperfuse the affected testis (1). However, reperfusion of ischemic tissue may cause further damage to the testes (2). It has been demonstrated that ischemia reperfusion (IR) injury in many organs is associated with recruitment and activation of neutrophils, proinflammatory cytokine generation, mitochondrial dysfunction and generation of reactive oxygen species (ROS) (3). These events often result in apoptosis of cells in the affected organ (4). Many studies recently reported loss of germ cells and disruption of the seminiferous epithelium after IR injury of the testis (5). In laboratory animal models of IR of the testis, permanent loss of spermatogenesis has been shown to be due to germ cell-specific apoptosis (6). Previously, several agents such as vitamin E, and erythropoietin were used to treat testicular IR injury (7, 8). Their beneficial effects have been shown to be due to their ability to abrogate oxidative stress in testicular IR.

Ghrelin which is primarily produced and secreted by the stomach has been implicated in a wide range of physiological and pathophysiological activities (9). The possible involvement of ghrelin in the protection of numerous tissues against IR injury has been shown (10). 

Expression of the functional ghrelin receptor, the GHS-R type 1a, has been revealed in Sertoli and Leydig cells (11). Hence, involvement of ghrelin in testicular functions is rational. Previously, it was shown that ghrelin modulates testicular germ cells apoptosis and proliferation in adult normal rats (12). On the other hand, functional roles of proinflammatory cytokines particularly tumor necroses factor (TNF)-α and interleukin (IL)-1β in the mammalian testis have been documented (6). Generally it is believed that TNF-α and IL-1β are increased after IR in testis and some model systems (1, 13). However, the role of ghrelin in the testicular germ cell apoptosis, proliferation and proinflammatory cytokine generation in testicular IR injury has not been yet delineated. 

Therefore, the present study was designed to examine the impact of ghrelin in bcl-2-associated X protein (BAX) and proliferating cell nuclear antigen (PCNA) expressions and its effect on the cytokines including TNF-*α*, IL-1β and interleukin (IL)-6 in testicular IR.

## Materials and methods

**Animals**

Male Wistar rats weighing 220-250 gr were housed in temperature- controlled room (24±1^o^C) on 12 hr light/dark cycle with free access to food and water. Animals were treated humanely and all procedures complied with the standards for care and use of animal subjects as stated in the Guide for the Care and Use of Laboratory Animals. The Medical Laboratory Animal Management Committee of Lorestan University of Medical Sciences approved all experiments. This study was performed (Jun-Aug, 2012) in Razi Herbal Medicines Research Center, Department of Physiology, School of Medicine, Lorestan University of Medical Sciences, Khorramabad, Iran.

**Experimental groups**

In an experimental study, 45 animals were selected for the study and divided randomly into 3 groups, each containing 15 rats. Group 1 was the control group (C) and animals in this group underwent sham operation without the application of the torsion. Group 2, the torsion/detorsion plus normal saline (T/D-S) group which underwent 1 hr of testicular torsion followed by 4 hr, 1 and 7 days of detorsion. 

The animals in this group received saline intraperitonally15 min before detorsion. Group 3, ghrelin treated group (T/D-G) was the test group which received ghrelin (40 nmol) 15 min before the testicular detorsion. The dose of the drug was chosen from the reports of the pilot study conducted in our laboratory. Torsion and detorsion times was the same as group 2. In all 3 groups, five rats from each group were killed upon diethyl ether anesthesia (May & Baker Ltd., Dagenham, England) by decapitation on 4 hr, 1 and 7 days after detorsion and unilateral orchiectomy was performed for biochemical and immunohistochemical examination.

**Surgical procedure**

Surgical torsion was carried out as described by Hamed *et al* (8). The animals were generally anesthetized with intraperitoneal injection of ketamine HCl (75 mg/kg) and xylazine (8 mg/kg) and the surgical operation described below was performed. The skin of scrotal area was shaved and then prepared with 10% povidone iodine solution. A right-sided midscrotal vertical incision was performed for access to testis. Torsion was created by twisting the right testes 720 in counterclockwise direction and maintained by fixing the testes to scrotum with a 4-0 silk suture passing through the tunica albuginea and dartos. 

After 1 hour of ischemia the suture was removed and right testes was detorted and replaced into scrotum for 4 hr, 1 and 7 days of reperfusion. During sham operation the right testes was brought through the incision and then replaced without twisting, and a silk suture was placed through the tunica albuginea. After each surgical intervention, the incision was closed using silk sutures.

**Bax and PCNA immunostaining**

Immunocytochemical analysis was carried out to identify BAX and PCNA expressions in the testicular germ cells. 3 lm dewaxed and rehydrated sections were immersed in target retrieval solution (pH= 9.0) and boiled water bath for 20 min at 98^o^C to delivered unmasked antigens. The sections were then dipped in 3% H_2_O_2_ for 10 min to block endogenous peroxidase and nonspecific background staining was blocked by incubating the sections for 3 min in 1% normal rabbit serum. 

The slides were incubated with the following antibodies: polyclonal rabbit antibody against BAX protein (dilution 1:40; Dako Corporation Carpinteria, CA, USA), mouse monoclonal anti-PCNA (Clone PC10; dilution 1:100; Dako, Glostrup, Denmark) followed by rinsing in TBS. Thereafter, all sections were incubated with biotinylated secondary antibody (Envision kit: Dako, Glostrup, Denmark) at room temperature for 30 min. The preparations were incubated with liquid diaminobenzidine tetrahydrochloride substrate (DAB, ready to use) for 10 min and were counterstained with Mayer’s hematoxylin, then were dehydrated, and mounted in Diatex at the end of processing. Negative control slides in the absence of primary antibody were also included.

**Reagent and drugs**

Expressions of BAX and PCNA were detected using available standard immunohistochemical kits provided from Dako Company (Glostrup, Denmark). Ghrelin was purchased from Tocris Cookson Ltd. (Bristol, UK). Rat IL-1β (BMS630), IL-6 (BMS625) and TNF-α (BMS622) Platinum ELISA kits were all obtained from eBioscience Company (Austria).

**Measurement of cytokines**

Each testis, was homogenized in 5 ml of 0.9% NaCl containing 0.5%Triton X100. The homogenate was centrifuged at 3000 gr for 15 min at 4^o^C and supernatant fluid was collected for quantitative detection of IL-1β, IL-6 and TNF-α by rat specific ELISA kit (eBioscience, Austria). The following limit of detection was observed for IL-1β, IL-6 and TNF-α respectively: sensitivity (4, 12, 11 pg/ml), intra assay variability (<10%), and inter-assay variability (<10%). Protein concentration of the supernatants was determined using Bradford method. Final concentration of IL-1β, IL-6 and TNF-α expressed per mg tissue protein.

**Statistical analysis**

The Statistical Package for the Social Sciences, version 16.0 (SPSS Inc, Chicago, Illinois, USA) was used to analyze the data. Tukey test was used after ANOVA to determine statistical differences among all of the groups. Data are presented as mean±SEM, and differences were considered to be significant at p<0.05.

## Results

**Figure 1:**
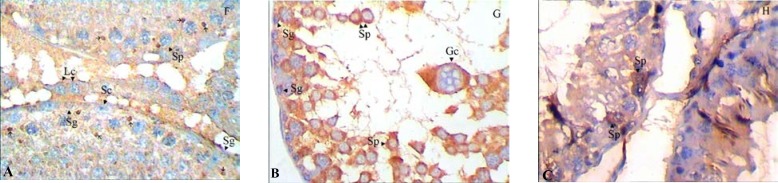
Immunohistochemical expression of germ cells applying polyclonal antibody against bax (A-C).

**Figure 2:**
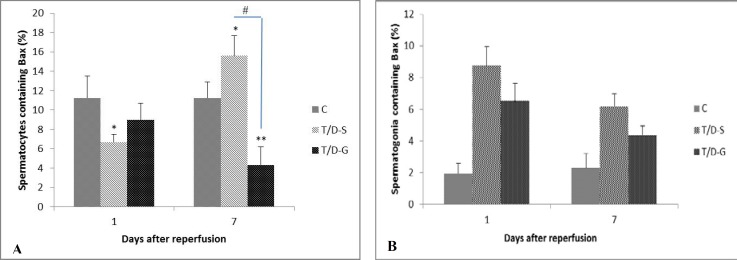
The mean percentages of spermatocytes (A) and spermatogonia (B) immunosatined against BAX substance in C, T/D-S and T/D-G groups on days 1 and 7 after reperfusion. Bars represent mean±SEM. Comparison among groups was made using one-way ANOVA followed Tukey’s post hoc test. All means marked * (p<0.05), ** (p<0.001) significantly differ from control group. Means marked with ^#^ (p<0.05) shows significantly difference between T/D-S and T/D-G groups.

In different groups, the brown intensive cytoplasmic stained cells for BAX protein indicates apoptosis in the spermatogonia and spermatocytes (Figure 1). The quantitative results of immunostaining in these cells are showed in figure 2. The number of cells exhibiting Bax expression was much higher in spermatocytes than the spermatogonia with no reaction in Sertoli cells (Figure 2). The percentage of spermatocytes containing BAX was significantly increased in T/D-S group versus control group on day 7 (p=0.041) (Figure 2A). After being treated by ghrelin, the population of immunoreactive cells against BAX in the spermatocytes on day 7 after reperfusion significantly decreased when compared to T/D-S animals (p=0.024). 

**Figure 3:**
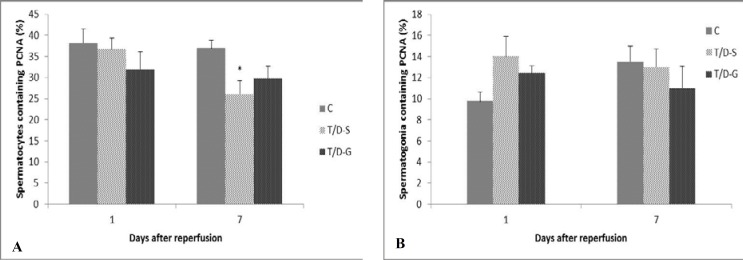
Percentages of spermatocytes (A) and spermatogonia (B) containing PCNA in all experimental groups calculated at 1 and 7 days after reperfusion. Bars represent mean±SEM. Comparison among groups was made using one-way ANOVA followed Tukey’s post hoc test. All means marked with * (p<0.05) significantly differ from control group.

BAX was expressed in 15.6±2.1 and 4.32±0.9% of T/D-S and T/D-G spermatocytes, respectively. After treatment with ghrelin, the positive cells showed a significant decline indicating that ghrelin has some protective effects on testes with ischemia injury. When the data was expressed about the spermatogonia, similar trend in the BAX positive cell content alterations were observed in response to the injection of ghrelin (Figure 2B). However, the effect of this hormone on down-regulating the expression of BAX in these cells was not statistically significant. When evaluated on the basis of the brown intensive nuclear stained cells against PCNA, it was shown that the PCNA expression in T/D-G group was slightly higher than T/D-S group only in spermatocytes on day 7 after reperfusion (Figure 3A). 

**Figure 4:**
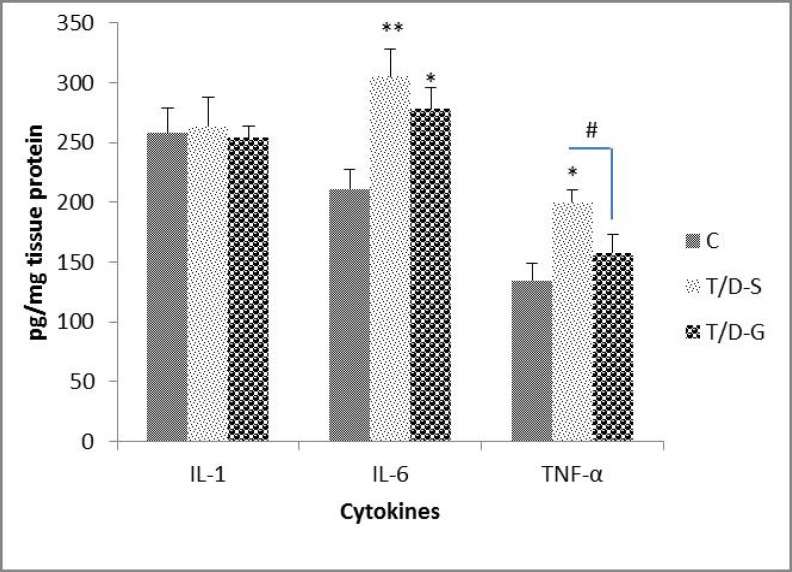
The mean levels of IL-1, IL-6 and TNFα in testicular tissue of C, T/D-S and T/D-G groups on 4 hours after reperfusion. All means marked * (p<0.05), ** (p<0.001) significantly differ from control group. Means marked with ^#^ (p<0.05) shows significantly difference between T/D-S and T/D-G groups. Data of figure 4 is shown in table I

This suggests that ghrelin could not significantly up-regulate the PCNA expression in injured testicular tissue. The number of positive cells against PCNA in the three groups is shown in figure 3. Testicular levels of cytokines (IL-1β, IL-6 and TNF-α) are depicted in figure 4. TNF-α and IL-6 in rats subjected to I/R injury were significantly higher than in the control animals (p=0.018 and p=0.0087, respectively). However, the animals in the T/D-S group tended to show mild increment in IL-1β. Administration of ghrelin significantly attenuated the testicular TNF-α and IL-6 levels compared with the untreated animals (p=0.033), but had no significant effect on the level of IL-1.

**Table I:** The mean levels of IL-1, IL-6 and TNFα in testicular tissue of C, T/D-S and T/D-G groups on 4 hours after reperfusion

**Groups**	**IL-1β**	**IL-6**	**TNF-α**
Control	258.67 ± 20.17	210.94 ± 16.77	134.65 ± 14.4
T/D-S	263.62 ± 24.65	305.4 ± 22.8**	200.49 ± 9.7*
T/D-G	254.12 ± 9.6	278.28 ± 18.1*	157.5 ± 15.19#

* (p<0.05),

** (p<0.01) significantly differ from control group. Means marked with

# (p<0.05) shows significantly difference between T/D-S and T/D-G groups.

(A): Control; The germ cells present immunolabeling for BAX primarily in spermatocytes (arrows) showing no evidences in spermatogonia. (B): Saline on day 7; Intensive BAX cytoplasmic expression in high population of spermatocytes, whereas the spermatogonia are more resistant to anti-BAX protein and therefore to apoptosis. (C): Ghrelin on day7; only few spermatocytes are showing cytoplasmic deep-brown staining against apoptotic marker (A-C) (400×).

Gc: Giant cell

Lc: Leydig cell

Sc: Sertoli cell

Sg: Spermatogonia

Sp: Spermatocyte

## Discussion

The data presented here are the first to show that ghrelin acts on germ cell proliferation and apoptosis in the rat testis after IR. Inhibitory effect of ghrelin on BAX expression was observed. Apoptosis maintains testicular tissue homeostasis in normal condition. Several studies have shown that ghrelin has anti-apoptotic and protective effects on various cell types subjected to IR injury (10). IR-induced germ cell-specific apoptosis is mainly mediated through an increase in ROS in the testis (5). There are two sources of ROS in post-ischemic tissues. One source of ROS is the hypoxanthine-xanthine oxidase reaction (2). Ischemia causes an increase in intracellular hypoxanthine as a result of ATP breakdown and then, during reperfusion, xanthine oxidase converts hypoxanthine to uric acid plus large quantities of superoxide radicals in the presence of oxygen. Neutrophil recruitment is the other source of ROS production (5). 

Neutrophils transmigrate through the endothelium into the interstitium of the testis and then release factors such as ROS that may directly cause apoptosis in germ cells (6). In addition current data suggest that ROS produced by the recruited neutrophils perturb Bcl-2 family members in the germ cells and thus initiate apoptosis (1). When ROS generation exceeds the defense mechanism’s capacity to control, oxidative stress is generated and contributes to reversible or irreversible cell injury (14). Antioxidant treatment, which enhances the endogenous antioxidant defense systems within cells, can inhibit a variety of the apoptotic pathways (15). 

In previous study we have demonstrated the antioxidant properties of ghrelin in the rat testis and we indicated that the impact of ghrelin pretreatment on epididymal sperm content and quality after IR injury may be due to its antioxidant ability (12). So, in this study ghrelin might suppress the apoptosis of germ cells through its antioxidant properties. Evaluation of the amount of PCNA showed that ghrelin slightly increased the PCNA levels only in spermatocytes on day 7 after reperfusion. Reduction in BAX substance level consistent with increased PCNA expression in the T/D-G animals on day 7 may contributes to regeneration of seminiferous tubules germ cells, and therefore helps to repair disrupted spermatogenesis.

In the present study, detection of cytokines IL-1β, TNF-α and IL-6 via ELISA analysis showed an increase in TNF-α and IL-6 and a trend upward for IL-1β after IR. Our findings are consistent with previous data that have demonstrated an increase in TNF-α expression after reperfusion of the testis (1, 16). Although up-regulation of the IL-1β also has been associated with IR in testes and other model systems, not significant increase of this cytokine was observed in T/D group in our experiment (1). The reason for this discrepancy cannot fully identify. Although previous studies have shown that administration of ghrelin reduces the production of pro-inflammatory cytokines in numerous organs, this is the first in vivo investigation indicates that ghrelin suppresses TNF-α and IL-6 after IR injury in testes (17). Most data implicate that ghrelin has anti-inflammatory effects through regulating the transcription and mRNA expression of pro-inflammatory cytokines (18). 

Ghrelin receptors are expressed in T lymphocytes, monocytes and macrophages where ghrelin inhibits the production of IL-1β, TNF-α and IL-6 (19). Interstitial testicular macrophages have been described a possible source of TNF-α. TNF-α is a multifunctional cytokine with effects not only in the proinflammatory response but in immunoregulatory responses, and apoptosis (6). There are some mechanisms that can be attributed for TNF-α induced apoptosis in the testis. TNFα may be acting upon Sertoli and Leydig cells contained the mRNA for TNFR1 (20). The type 1 TNF-α receptors are known for their ability to induce cell death via the activation of caspases and cell death (15). Likewise, Lysiak *et al* suggested that the increase in TNF-α may lead to the activation of NFκB in Sertoli cells and may influence germ cell apoptosis by increasing FasL expression in Sertoli cells (21). 

The inhibitory effect of TNF-α on testosterone secretion from Leydig cells has been reported in porcine and murine Leydig cells (6). On the other hand, involvement of testosterone in the control of germ cells apoptosis, beside cell proliferation and differentiation during spermatogenesis is well documented (11). Taken together it is possible to speculate that the inhibitory effect of ghrelin on the TNF-α expression, beside its antioxidant properties is another way to suppress the germ cell apoptosis by ghrelin in the present study. Previously it has been indicated that ghrelin abrogates TNF-α induced anti-proliferative and pro-apoptotic effects in human adipocytes and intestinal epithelial cells (22). There is now good evidence that I/R and/or inflammation leads to a significant increase in the expression of IL-6 in many organs including the brain, hind limb, and gut and kidney (13, 23-25). 

There is no literature reference on the possible role of IL-6 in the testis IR injury and this deserves to be investigated. However, the role of endogenous IL-6 in the tissue injury and inflammation associated with ischemia/ reperfusion (IR) is controversial. For instance, Herrmann *et al* suggested that endogenous IL-6 protects the brain against I/*R*-injury while, it was showed that an enhanced expression of IL-6 contributes to gut and renal IR injury (26-28). The IL-6 is a pleiotropic cytokine regulates the expression of other cytokines including IL-1β and TNF-α, which in turn potently enhances the inflammatory response, and also forms part of a positive feedback cycle in which TNF-α stimulates IL-6 production (28). 

Patel *et al* confirmed that in the presence of endogenous IL-6, the degree of inflammation, and, hence, the formation of TNF-α and IL-1β, caused by renal I/R are significantly enhances. Furthermore, IL-6 increases polymorphonuclear neutrophil accumulation within the different tissues by causing amplification (positive feedback) of the formation of the proinflammatory cytokines TNF-α and IL-1β and/or of the expression of the adhesion molecule E-selection (6). Previously the ability of IL-6 in the increase of lipid peroxidation in the IR conditions has been demonstrated (25).

All of the above studies support the view that endogenous IL-6 enhances the degree of oxidative stress. Since ghrelin abolished the expression of IL-6 in the T/D-G group in this study, thus it is possible to postulate that ghrelin also may protect the testicular antioxidant defense system via IL-6 expression which is subsequently leads to reduction in germ cell apoptosis. Further investigation of this possibility is warranted.

## Conclusion

In conclusion, the results of the present study indicate that ghrelin, offers remarkable anti-inflammatory and anti-apoptotic effects in testicular ischemia reperfusion injury.
